# Supplementary material for: Two-dimensional multiferroic material of metallic p-doped SnSe
Source: Nat Commun. 2022 Oct 17;13:6130. doi: 10.1038/s41467-022-33917-2 (PMC9576753; doi:10.1038/s41467-022-33917-2)
Supplement: Supplementary file 1 — Supplementary Information [file 41467_2022_33917_MOESM1_ESM.pdf]

Supplementary Information

for

Two-dimensional multiferroic material of metallic p-doped SnSe

Ruofan Du[1#], Yuzhu Wang[1#], Mo Cheng[1], Peng Wang[1], Hui Li[1], Wang Feng[1], Luying Song[1], Jianping Shi[1*], Jun He[2*]

[1]The Institute for Advanced Studies, Wuhan University, Wuhan 430072, China

[2]Key Laboratory of Artificial Micro- and Nano-structures of Ministry of Education, School of Physics and Technology, Wuhan University, Wuhan 430072, China

*Address correspondences: jianpingshi@whu.edu.cn, He-jun@whu.edu.cn

[#]These authors contributed equally: Ruofan Du, Yuzhu Wang

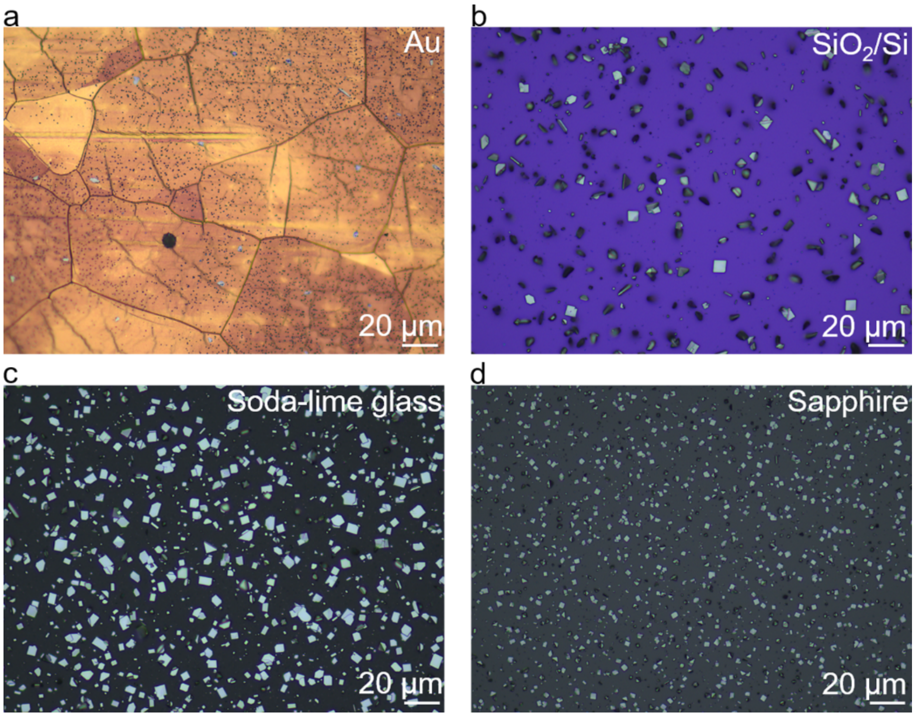

Supplementary Figure 1 | OM images of PVD-synthesized 2D SnSe nanosheets on different substrates (*e.g.*, Au foil, soda-lime glass, SiO_2/Si, and sapphire). Smaller and thicker SnSe nanosheets are obtained, suggesting the advantage of mica substrate for growing the large-domain and ultrathin SnSe.

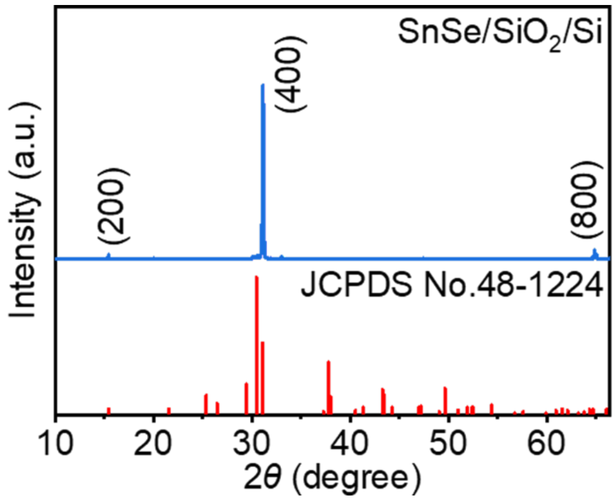

Supplementary Figure 2 | XRD characterization of transferred 2D SnSe nanosheets on SiO_2/Si, revealing its orthorhombic phase and high crystal quality.

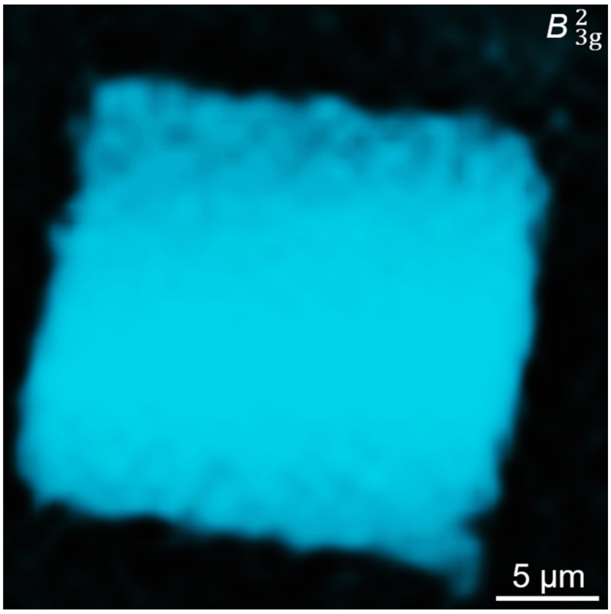

Supplementary Figure 3 | Raman intensity (B^{2}_{3g}) mapping image of a tetragonal SnSe nanosheet.

The rather uniform color contast shows its high thickness uniformity.

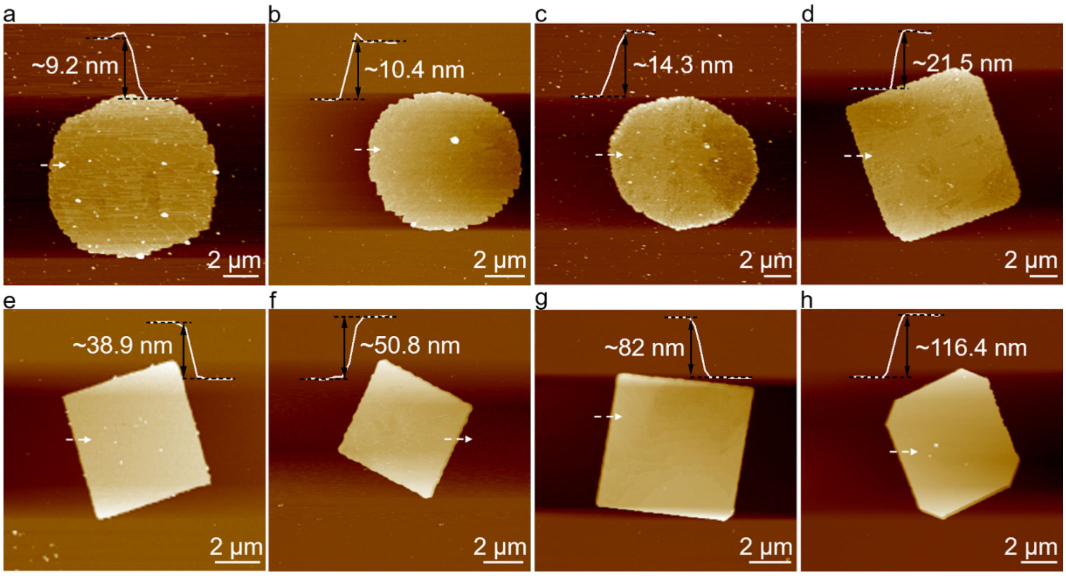

Supplementary Figure 4 | AFM images and corresponding height profiles analyses of as-grown 2D SnSe nanosheets with different thicknesses.

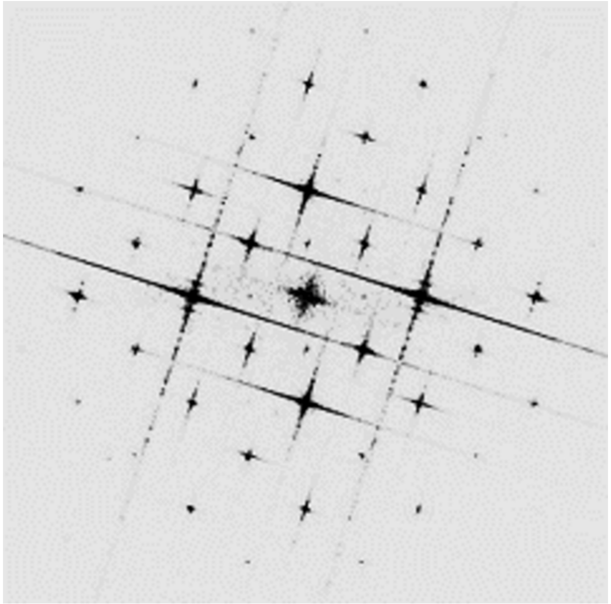

Supplementary Figure 5 | FFT pattern of a transferred tetragonal SnSe nanosheet, showing its single crystalline feature.

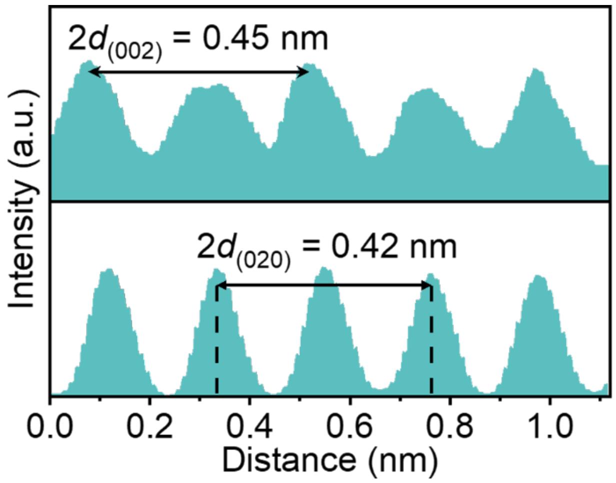

Supplementary Figure 6 | The STEM intensity line profiles along different planes, showing the lattice constant of ~2.1 Å, consistent with the (020) plane spacing of SnSe.

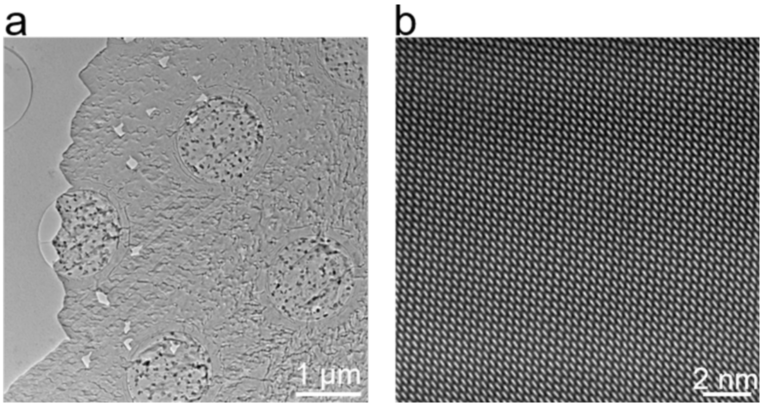

Supplementary Figure 7 | TEM characterization of a transferred circle SnSe nanosheet. a Low-magnification TEM image of a circle SnSe nanosheet. **b** Corresponding atomic-resolution TEM image.

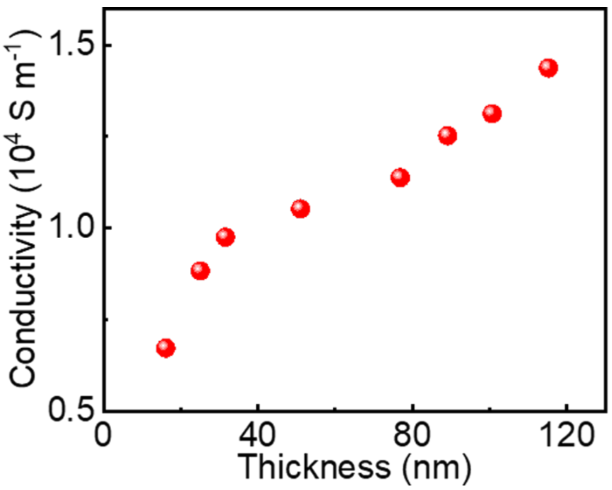

Supplementary Figure 8 | Thickness-depedent conductivity of PVD-synthesized 2D p-doped SnSe with the electrode direction along zigzag orientation.

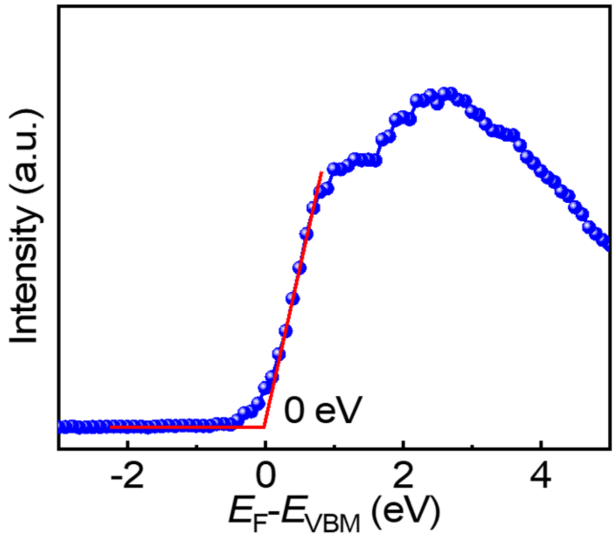

Supplementary Figure 9 | Valence band photoemission spectrum of PVD-synthesized 2D SnSe on mica. The intensive DOS near the Fermi level indicates its metallic feature.

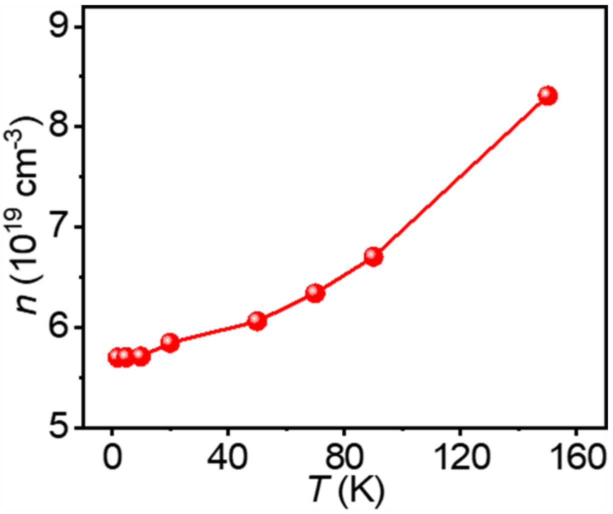

Supplementary Figure 10 | Temperature-dependent carrier concentration of 2D p-doped SnSe.

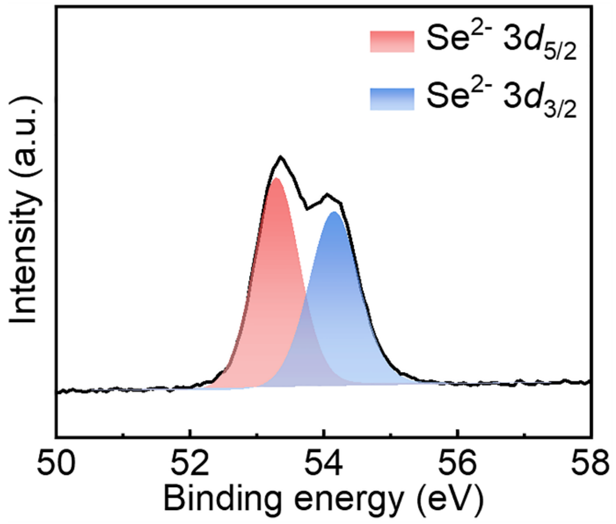

Supplementary Figure 11 | XPS spectrum of transferred 2D p-doped $SnSe_2$ nanosheets on SiO_2/Si.

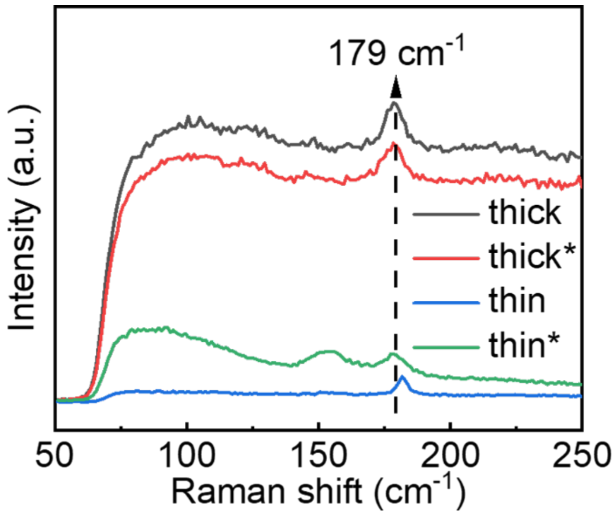

Supplementary Figure 12 | Raman characterizations of transfer flipped SnSe nanosheets, revealing the presence of $SnSe_2$ microdomains.

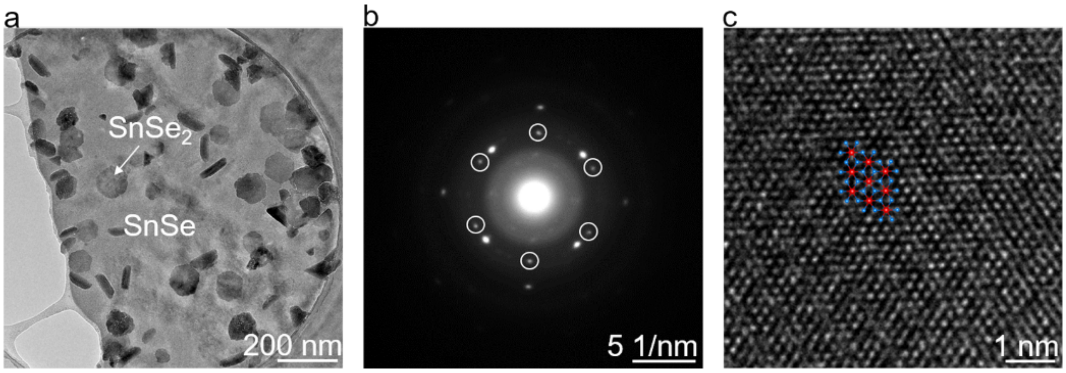

Supplementary Figure 13 | The determination of $SnSe_2$ microdomains by TEM. a Low-magnification TEM image of a p-doped SnSe nanosheet, showing the formation of $SnSe_2$ microdomains. **b** Corresponding SAED pattern captured from the $SnSe_2$ region. **c** Atomic-resolution TEM image obtained from the $SnSe_2$ area.

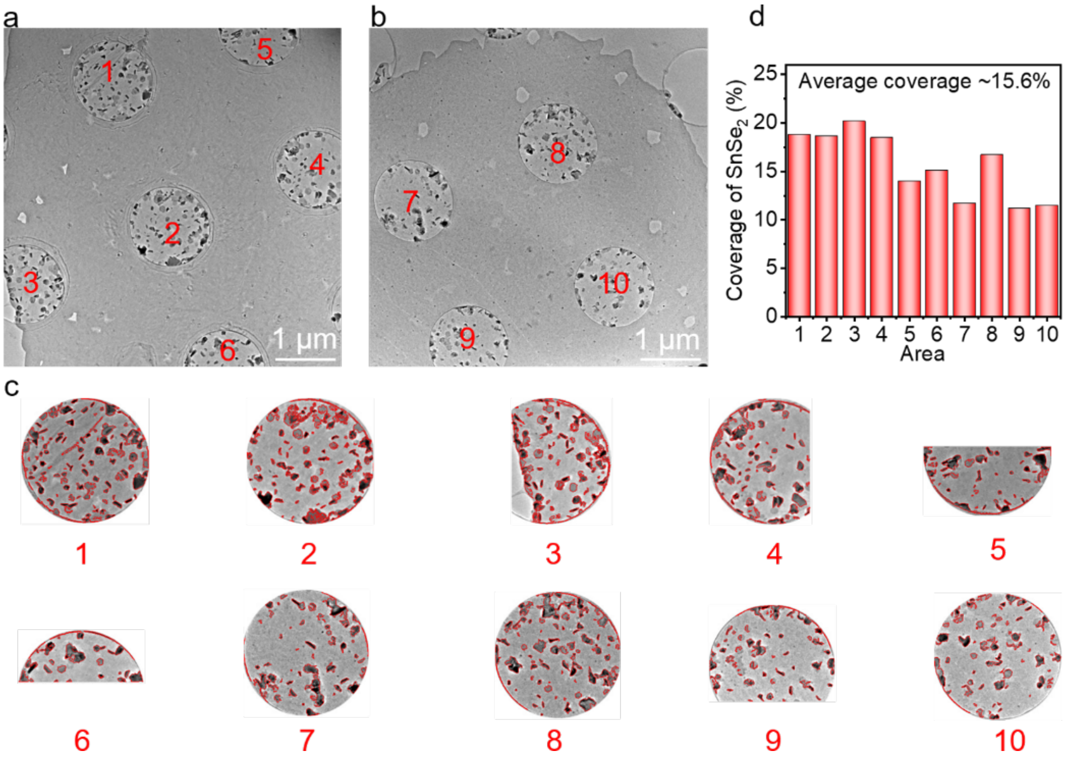

Supplementary Figure 14 | The coverage determination of $SnSe_2$ microdomains. a–c Low-magnification TEM images captured from different areas of p-doped SnSe. **d** Statistical distribution of the coverage of $SnSe_2$ microdomains.

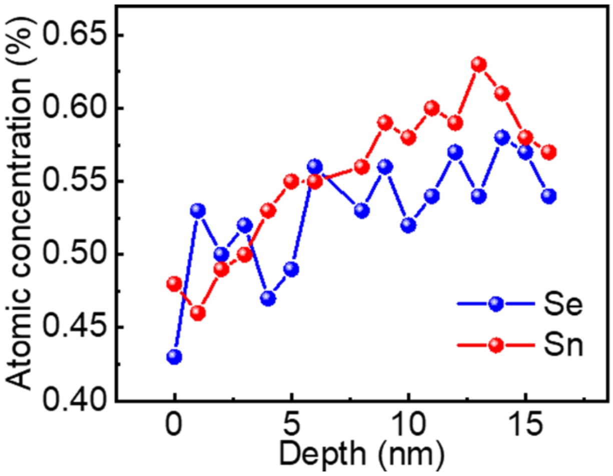

Supplementary Figure 15 | The XPS depth analysis of $SnSe/SnSe_2$ heterostructure with the thickness of ~16 nm. The thicknesses of $SnSe_2$ and SnSe are determined to be 2~4 nm and 14~12 nm, respectively.

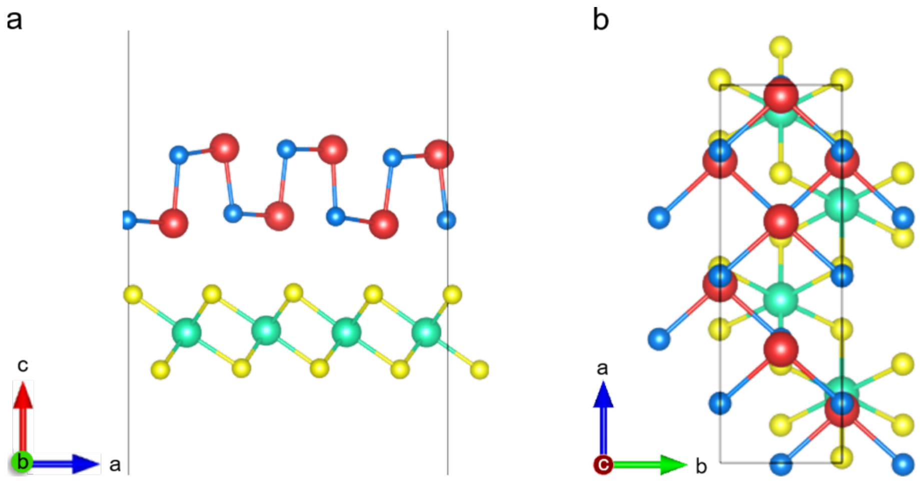

Supplementary Figure 16 | The atomic structure of SnSe/$SnSe_2$, which is constructed by stacking 1 × 3 supercell of SnSe on 1 × 2 supercell of $SnSe_2$. To minimize the lattice mismatch between the stacking layers, a rectangular unit cell of $SnSe_2$ is constructed.

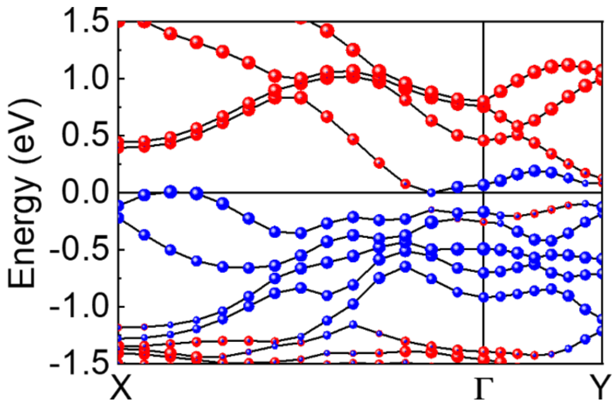

Supplementary Figure 17 | Calculated the band structure of $SnSe/SnSe_2$ by using DFT method with the HSE function. The Fermi level is crossed by several bands, indicative of its metallic feature.

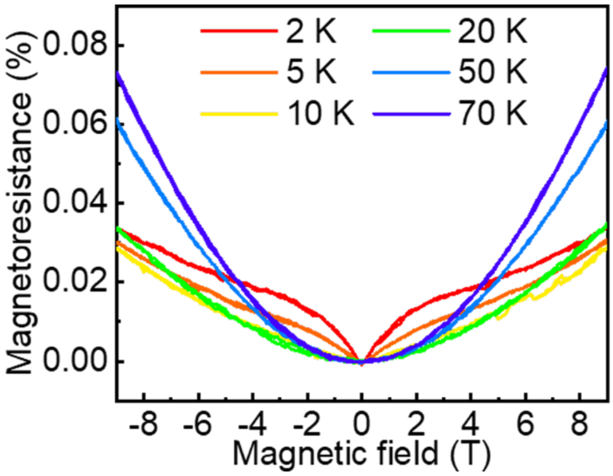

Supplementary Figure 18 | Temperature-dependent magnetoresistance of PVD-synthesized 2D metallic p-doped SnSe nanosheet (with the thickness of ~46 nm) with a parallel magnetic field. A sharp dip around zero magnetic field suggestes the appearance of weak antilocalization (WAL) effect.

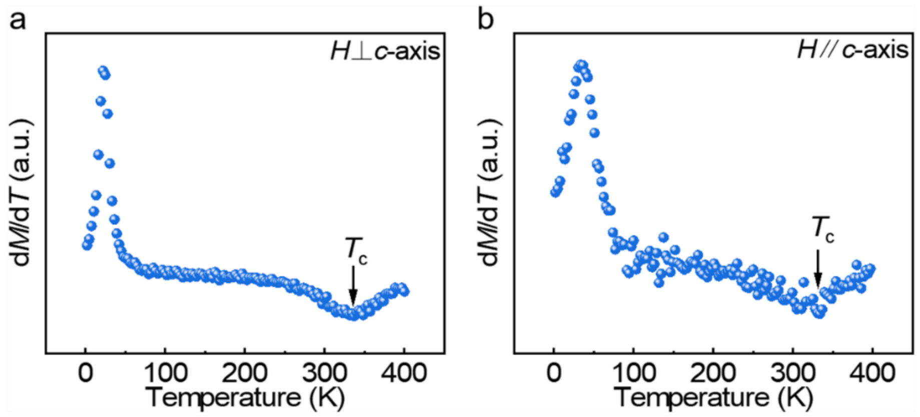

Supplementary Figure 19 | ZFC derivative curves of PVD-synthesized 2D p-doped SnSe, showing the Curie temperature of ~337 K. The magnetic field (100 Oe) is vertical and parallel to *c*-axis, respectively.

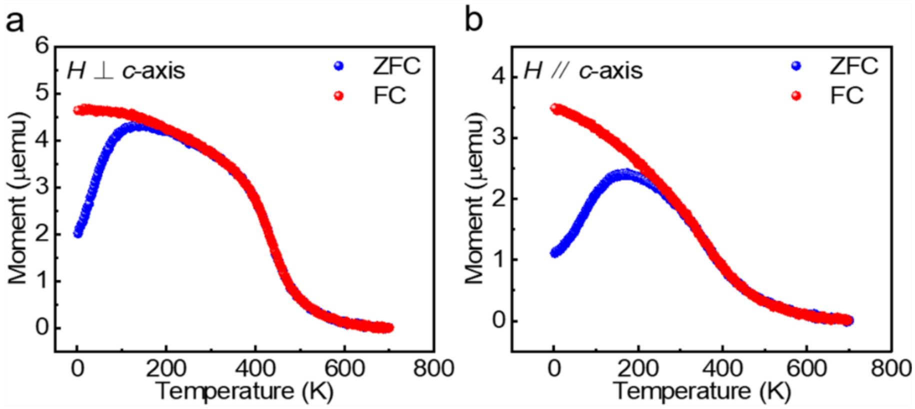

Supplementary Figure 20 | Temperature-dependent magnetic moment of 2D p-doped SnSe nanosheets with a vertical and parallel magnetic field at 100 Oe. The ferromagnetic order is disappeared at ~665 K, reconfirming the high temperature ferrimagnetic property of 2D p-doped SnSe.

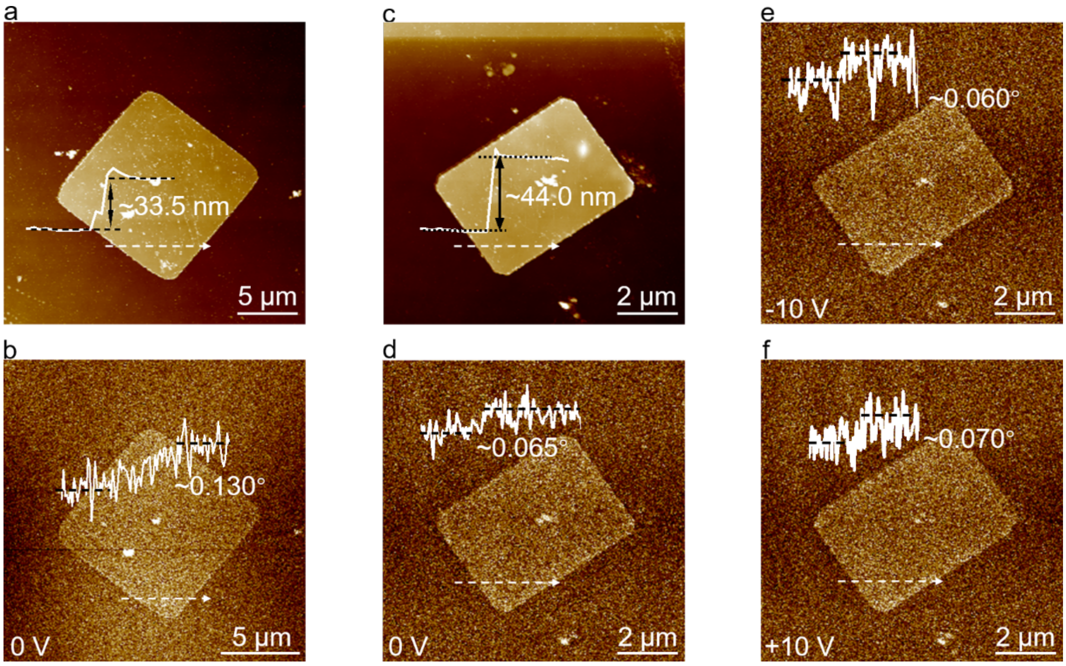

Supplementary Figure 21 | MFM measurements of PVD synthesized 2D p-doped SnSe with different thicknesses at room-temperature. a AFM image and corresponding height profile analysis of a p-doped SnSe nanosheet with the thickness of ~33.5 nm. **b** Corresponding MFM phase image. The phase deviation between p-doped SnSe and nonmagnetic SiO_2/Si substrate indicates the ferromagnetic order of p-doped SnSe. The single magnetic domain is obtained for p-doped SnSe. **c** AFM image and corresponding height profile analysis of a p-doped SnSe nanosheet with the thickness of ~44.0 nm. **d**−**f** Corresponding MFM phase images captured from different DC voltages.

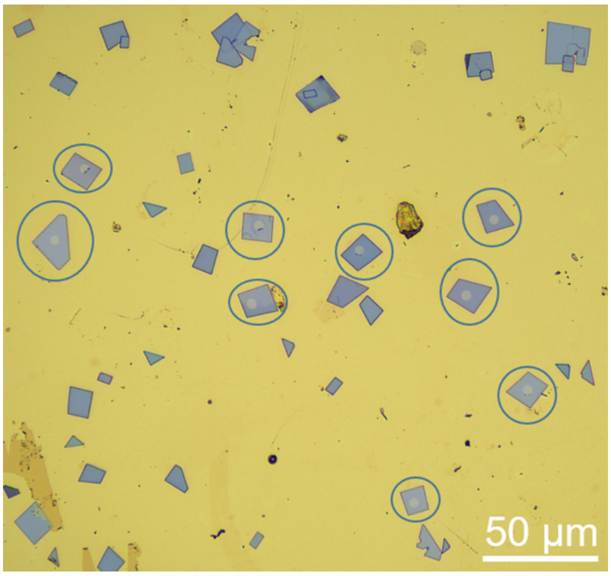

Supplementary Figure 22 | OM image of transferred 2D metallic p-doped SnSe nanosheets on Au/Si/ITO. The top Au electrodes are deposited on 2D p-doped SnSe nanosheets to avoid the local charge/ion accumulation.

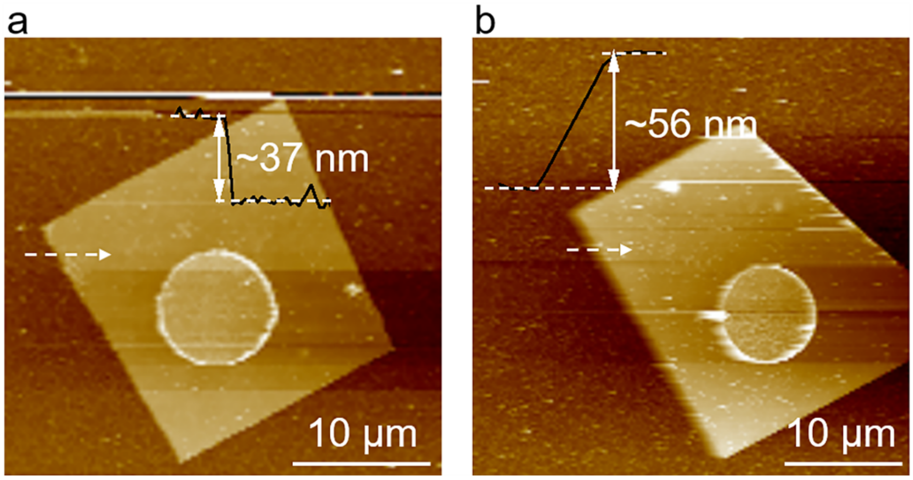

Supplementary Figure 23 | AFM images and corresponding height profile analyses of transferred tetragonal SnSe nanosheets with the top Au electrodes.

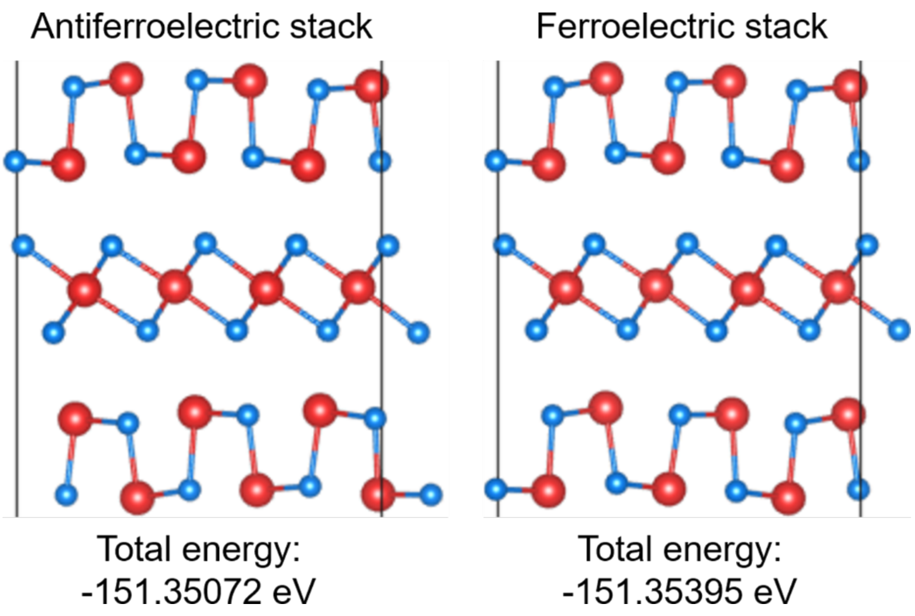

Supplementary Figure 24 | The energies of ferroelectric and antiferroelectric stacking of $SnSe/SnSe_2$. The much smaller energy of ferroelectric stack suggests its relatively high stable structure.

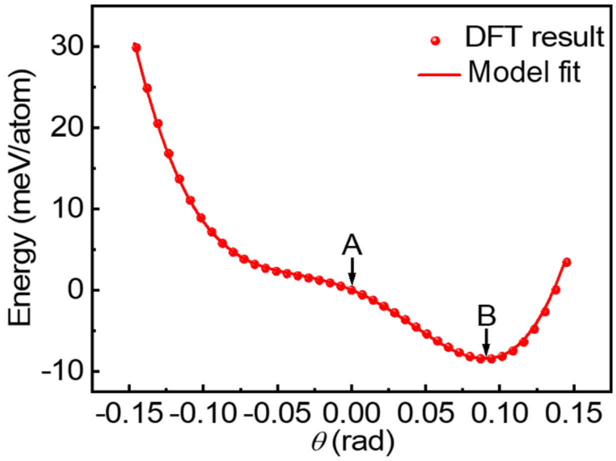

Supplementary Figure 25 | The single-well potential of SnSe/$SnSe_2$ interface. A and B represent the nonpolar and polar phase, respectively.

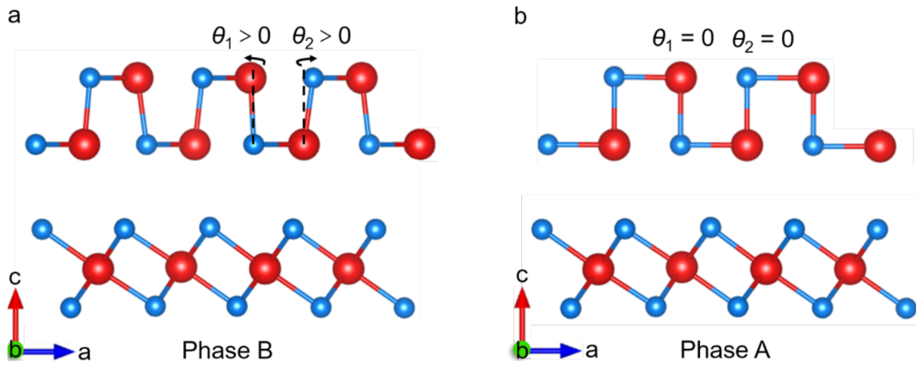

Supplementary Figure 26 | Schematic diagram of the distorted degenerate polar structure (B) and high symmetry nonpolar phase (A).

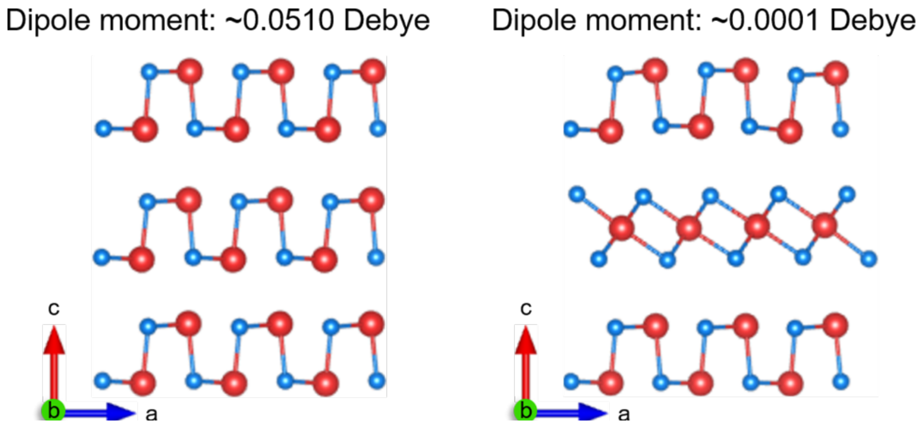

Supplementary Figure 27 | Atomic structures of trilayer SnSe and SnSe/$SnSe_2$. The dipole moments of 2D p-doped SnSe are calculated by using the bond-valence method and Debye equation. The small dipole moment value comparing with that of intrinsic SnSe indicates the weak ferroelectricity in 2D p-doped SnSe, consistent with the experimental results.

Table S1. The lattice constants of SnSe and $SnSe_2$

	a (Å)	b (Å)	γ (°)
$SnSe_2$	3.87	6.70	90
SnSe	4.29	4.40	90

Table S2. The remanence, coercivity, and saturation magnetism of 2D p-doped SnSe

Temperature (K)	In plane			Out of plane		
	Remanence (μemu)	Coercivity (T)	Saturation magnetism (μemu)	Remanence (μemu)	Coercivity (T)	Saturation magnetism (μemu)
300	2.2	0.009	123.4	24.0	0.020	134.1
5	107.5	0.018	528.7	3.6	0.020	113.2

Table S3. The refined atomic coordinates of SnSe and $SnSe/SnSe_2$

SnSe				$SnSe/SnSe_2$			
Atom	*x*	*y*	*z*	Atom	*x*	*y*	*z*
Sn1	0.9625	0.1465	0.3760	Sn1	0.98806	0.15033	0.37842
Sn2	0.4729	0.3098	0.4547	Sn2	0.48818	0.31479	0.46204
Sn3	0.9639	0.4794	0.3760	Sn3	0.98819	0.48503	0.38458
Sn4	0.4751	0.6431	0.4544	Sn4	0.48795	0.65147	0.46216
Sn5	0.9647	0.8128	0.3757	Sn5	0.98876	0.81461	0.37812
Sn6	0.4732	0.9763	0.4545	Sn6	0.48813	0.98514	0.45785
Sn7	0.2755	0.1385	0.0308	Sn7	0.99052	0.19134	0.24364
Sn8	0.7706	0.3044	0.1116	Sn8	0.49442	0.44078	0.24361
Sn9	0.2771	0.4715	0.0303	Sn9	0.98473	0.69343	0.24624
Sn10	0.7705	0.6379	0.1113	Sn10	0.49165	0.94319	0.24752
Sn11	0.2775	0.8052	0.0302	Sn11	0.21672	0.15078	0.02906
Sn12	0.7708	0.9708	0.1114	Sn12	0.71973	0.31766	0.10789
Sn13	0.8473	0.1338	0.1990	Sn13	0.21872	0.48400	0.02859
Sn14	0.3513	0.3052	0.2797	Sn14	0.71414	0.65096	0.10793
Sn15	0.8481	0.4677	0.1989	Sn15	0.22038	0.81708	0.02806
Sn16	0.3515	0.6383	0.2798	Sn16	0.72069	0.98321	0.10884
Sn17	0.8507	0.8005	0.1988	Se1	0.48804	0.33749	0.38567
Sn18	0.3530	0.9715	0.2798	Se2	0.98766	0.16837	0.45706
Se1	0.4721	0.3287	0.3772	Se3	0.48743	0.66481	0.38485
Se2	0.9727	0.1624	0.4539	Se4	0.98654	0.50460	0.46251
Se3	0.4739	0.6618	0.3768	Se5	0.48770	0.00242	0.38101
Se4	0.9736	0.4958	0.4539	Se6	0.98887	0.83798	0.45549
Se5	0.4719	-0.0043	0.3770	Se7	0.99234	0.02629	0.29057
Se6	0.9730	0.8288	0.4535	Se8	0.49135	0.10684	0.20087
Se7	0.7782	0.3246	0.0341	Se9	0.49116	0.2719	0.28719
Se8	0.2797	0.1576	0.1077	Se10	0.99368	0.36121	0.19938
Se9	0.7789	0.6583	0.0339	Se11	0.98926	0.52054	0.28828
Se10	0.2801	0.4910	0.1072	Se12	0.49181	0.61413	0.20181
Se11	0.7776	-0.0085	0.0341	Se13	0.4856	0.77314	0.2908
Se12	0.2797	0.8242	0.1071	Se14	0.98198	0.86323	0.20356
Se13	0.3392	0.3221	0.2019	Se15	0.72086	0.33676	0.02974
Se14	0.8413	0.1553	0.2773	Se16	0.21614	0.16854	0.10582
Se15	0.3407	0.6553	0.2020	Se17	0.71887	0.66984	0.02978
Se16	0.8415	0.4885	0.2774	Se18	0.21541	0.50174	0.1055
Se17	0.3392	0.9882	0.2019	Se19	0.71795	0.00376	0.03109
Se18	0.8421	0.8217	0.2772	Se20	0.2231	0.83381	0.10486

Table S4. The bond distances (Å) of SnSe and SnSe/$SnSe_2$

SnSe		$SnSe/SnSe_2$	
Sn1-Se2	2.76157	Sn1-Se2	2.78958
Sn1-Se5	2.95772	Sn1-Se5	2.90228
Sn2-Se1	2.75045	Sn2-Se1	2.71576
Sn2-Se2	2.89695	Sn2-Se2	2.89246
Sn3-Se1	2.95312	Sn3-Se1	2.89847
Sn3-Se4	2.76205	Sn3-Se4	2.76640
Sn4-Se3	2.75373	Sn4-Se3	2.73805
Sn4-Se4	2.89185	Sn4-Se4	2.88829
Sn5-Se3	2.95891	Sn5-Se3	2.92436
Sn5-Se6	2.75803	Sn5-Se6	2.75187
Sn6-Se5	2.75106	Sn6-Se5	2.72534
Sn6-Se6	2.89791	Sn6-Se6	2.89887
Sn7-Se8	2.72963	Sn7-Se7	2.74014
Sn7-Se11	2.88958	Sn7-Se8	2.84915
Sn8-Se7	2.75225	Sn7-Se9	2.84873
Sn8-Se8	2.92444	Sn7-Se10	2.73611
Sn9-Se7	2.89263	Sn8-Se9	2.71166
Sn9-Se10	2.73010	Sn8-Se10	2.85250
Sn10-Se9	2.74904	Sn8-Se11	2.84781
Sn10-Se10	2.86627	Sn8-Se12	2.72587
Sn11-Se9	2.90041	Sn9-Se11	2.72542
Sn11-Se12	2.72947	Sn9-Se12	2.88032
Sn12-Se11	2.74583	Sn9-Se13	2.85917
Sn12-Se12	2.86616	Sn9-Se14	2.70379
Sn13-Se14	2.78202	Sn10-Se7	2.85214
Sn13-Se17	2.85757	Sn10-Se8	2.71942
Sn14-Se14	2.88963	Sn10-Se13	2.71851
Sn14-Se13	2.75923	Sn10-Se14	2.82050
Sn15-Se13	2.85517	Sn11-Se16	2.72306
Sn15-Se16	2.78815	Sn11-Se19	2.89887
Sn16-Se15	2.75924	Sn12-Se15	2.77357
Sn16-Se16	2.88873	Sn12-Se16	2.90237
Sn17-Se15	2.84840	Sn13-Se15	2.90312
Sn17-Se18	2.78526	Sn13-Se18	2.72836
Sn18-Se17	2.76268	Sn14-Se17	2.77337
Sn18-Se18	2.88614	Sn14-Se18	2.91895
		Sn15-Se17	2.89187
		Sn15-Se20	2.72336
		Sn16-Se19	2.76133
		Sn16-Se20	2.92628
